# ARDS Patients Exhibiting a “Hyperinflammatory Anasarca” Phenotype Could Benefit From a Conservative Fluid Management Strategy

**DOI:** 10.3389/fmed.2021.727910

**Published:** 2021-08-25

**Authors:** Chun-yan Xing, Wen-bin Gong, Yan-Na Yang, Xin-jie Qi, Shi Zhang

**Affiliations:** ^1^Department of Pulmonary and Critical Care Medicine, Jinan Central Hospital, Cheeloo College of Medicine, Shandong University, Jinan, China; ^2^Department of Pulmonary and Critical Care Medicine, Jinan Central Hospital Affiliated to Shandong First Medical University, Jinan, China

**Keywords:** ARDS, conservative fluid management, liberal fluid management, phenotype, hyperinflammatory anasarca

## Abstract

**Object:** The fluid management strategy in ARDS is not very clear. A secondary analysis of RCT data was conducted to identify patients with ARDS benefitting from a conservative strategy of fluid management.

**Methods:** The data of this study were downloaded from the ARDS network series of randomized controlled trials (Conservative Strategy vs. Liberal Strategy in 2006). Based on the clinical feature of patients, within the first 24 h after admission, clustering was performed using the *k*-means clustering algorithm to identify the phenotypes of ARDS. Survival was analyzed using the Kaplan-Meier survival analysis to assess the effect of the two fluid management strategies on the 90-day cumulative mortality. Categorical/dichotomic variables were analyzed by the chi-square test. Continuous variables were expressed as the mean and standard deviation and evaluated through a one-way ANOVA. A *P*-value < 0.05 was defined as the statistically significant cut-off value.

**Results:** A total of 1,000 ARDS patients were enrolled in this unsupervised clustering research study, of which 503 patients were treated with a conservative fluid-management strategy, and 497 patients were treated with a liberal fluid-management strategy. The first 7-day cumulative fluid balance in patients with the conservative strategy and liberal strategy were −136 ± 491 ml and 6,992 ± 502 ml, respectively (*P* < 0.001). Four phenotypes were found, and the conservative fluid-management strategy significantly improved the 90-day cumulative mortality compared with the liberal fluid-management strategy (HR = 0.532, *P* = 0.024) in patients classified as “hyperinflammatory anasarca” phenotype (phenotype II). The characteristics of this phenotype exhibited a higher WBC count (20487.51 ± 7223.86/mm^3^) with a higher incidence of anasarca (8.3%) and incidence of shock (26.6%) at baseline. The furthermore analysis found that the conservative fluid management strategy was superior to the liberal fluid management strategy in avoiding superinfection (10.10 vs. 14.40%, *P* = 0.037) and returned to assisted breathing (4.60 vs. 16.20%, *P* = 0.030) in patients classified as “hyperinflammatory anasarca” phenotype. In addition, patients with other phenotypes given the different fluid management strategies did not show significant differences in clinical outcomes.

**Conclusion:** Patients exhibiting a “hyperinflammatory anasarca” phenotype could benefit from a conservative fluid management strategy.

## Background

Acute respiratory distress syndrome (ARDS) refers to acute inflammatory injury of the lung, disruption of the alveolar–capillary barrier and the formation of non-cardiogenic, protein-rich pulmonary oedema (1–4). A conservative fluid management strategy could improve the anasarca and oxygenation index (PaO2/FiO2). In addition, initiating treatment to reduce pulmonary oedema as early as possible could decrease the risk of superinfection (5, 6).

Although lung failure alone can be lethal, death in patients with acute lung injury is usually due to the failure of the non-pulmonary organs. Conservative fluid management strategies may lead to lower intravascular volume and perfusion (7, 8). Wiedemann et al. performed a randomized controlled trial (RCT) to compare conservative and liberal fluid-management strategies in ARDS (9). The results indicated that although the conservative strategy of fluid management improved lung function and shortened the duration of mechanical ventilation, a conservative strategy could not improve the mortality of ARDS. This suggested that not all ARDS patients need dehydration therapy for the improvement of lung function, and the sufficient effective circulating blood volume could also be took into account in parts of ARDS patient. Therefore, the fluid management strategy for ARDS is not very clear.

Artificial intelligence (AI) has found its way into clinical studies in the era of big data. Meanwhile, as increasing number of ARDS clinical trials data is open to public, secondary analysis on these combined datasets provide a powerful way of finding solution to clinical questions with a new perspective (10, 11). When combined with machine learning informatics and clinical trials data, the result will be the development of a precision form of personalized treatment applied to ARDS, which could be a promising way to explore the precise fluid management for specific ARDS population (12).

Based on this clinical problem, the hypothesis for identification of the specific ARDS patients who could benefit from conservative fluid management would be tested through a secondary analysis on RCT data from Wiedemann et al. using a machine learning algorithm (unsupervised clustering).

## Methods

The data of this study were downloaded from the ARDS network series of randomized controlled trials (Conservative Strategy vs. Liberal Strategy in 2006) (9). A total of 1,000 ARDS patients participated in this study.

### Screening Features for Unsupervised Clustering

Clinical features of ARDS patients were obtained before the start of treatment with a conservative strategy or liberal strategy within the first 24 h after admission. If missing data for a certain feature or sample is more than 5% then we will leave that feature. The other missing data (<5%) were estimated by multiple imputations through the R package, following the process described by Zhang with minor modifications (13). The mice R package conducted three main steps: (1) imputation, (2) analysis, and (3) pooling for missing data. The imputation step identified the characteristic of missing data; then the analysis step provided the predictive mean matching of missing data through modular approach; finally, the pooling step filled up the missing data based on 1,000 imputations iterations (13–15).

To screen suitable clinical features for clustering analysis, we attempted to train several classifiers from scratch. The clear separations and significant statistical results (*P* < 0.05) were utilized as the criterion for the identification of suitable clinical features for the best classification model.

### Statistical Methods

Clustering was performed using the *k*-means clustering algorithm implemented in R (*k*-means package). The best classifications were selected based on clear separations of the consensus heatmaps.

Survival was analyzed using the Kaplan-Meier survival analysis to assess the effect of the two fluid management strategies on the 90-day cumulative mortality. Categorical/dichotomic variables were analyzed by the chi-square test. Continuous variables were expressed as the mean and standard deviation and evaluated through a one-way ANOVA.

A *P*-value < 0.05 was defined as the statistically significant cut-off value.

### Software

All the analyses in this study were conducted using R 4.0.3.

## Results

### Patients

A total of 1,000 ARDS patients were enrolled in this unsupervised clustering research study, of which 503 patients were treated with a conservative fluid-management strategy, and 497 patients were treated with a liberal strategy fluid-management strategy. The first 7-day cumulative fluid balance in patients with the conservative strategy and liberal strategy were −136 ± 491 ml and 6,992 ± 502 ml, respectively (*P* < 0.001).

### Characteristics for Unsupervised Clustering

After multiclustering, the Acute Physiology and Chronic Health Evaluation III (APACHE III) score, PaO2, central venous pressure (CVP), predicted body weight (PBW), white blood cell count (WBC), platelet count, and the presence or absence of shock and anasarca were finally enrolled in further unsupervised clustering analysis.

### Clinical Outcomes of Phenotypes

**Figure 1:**
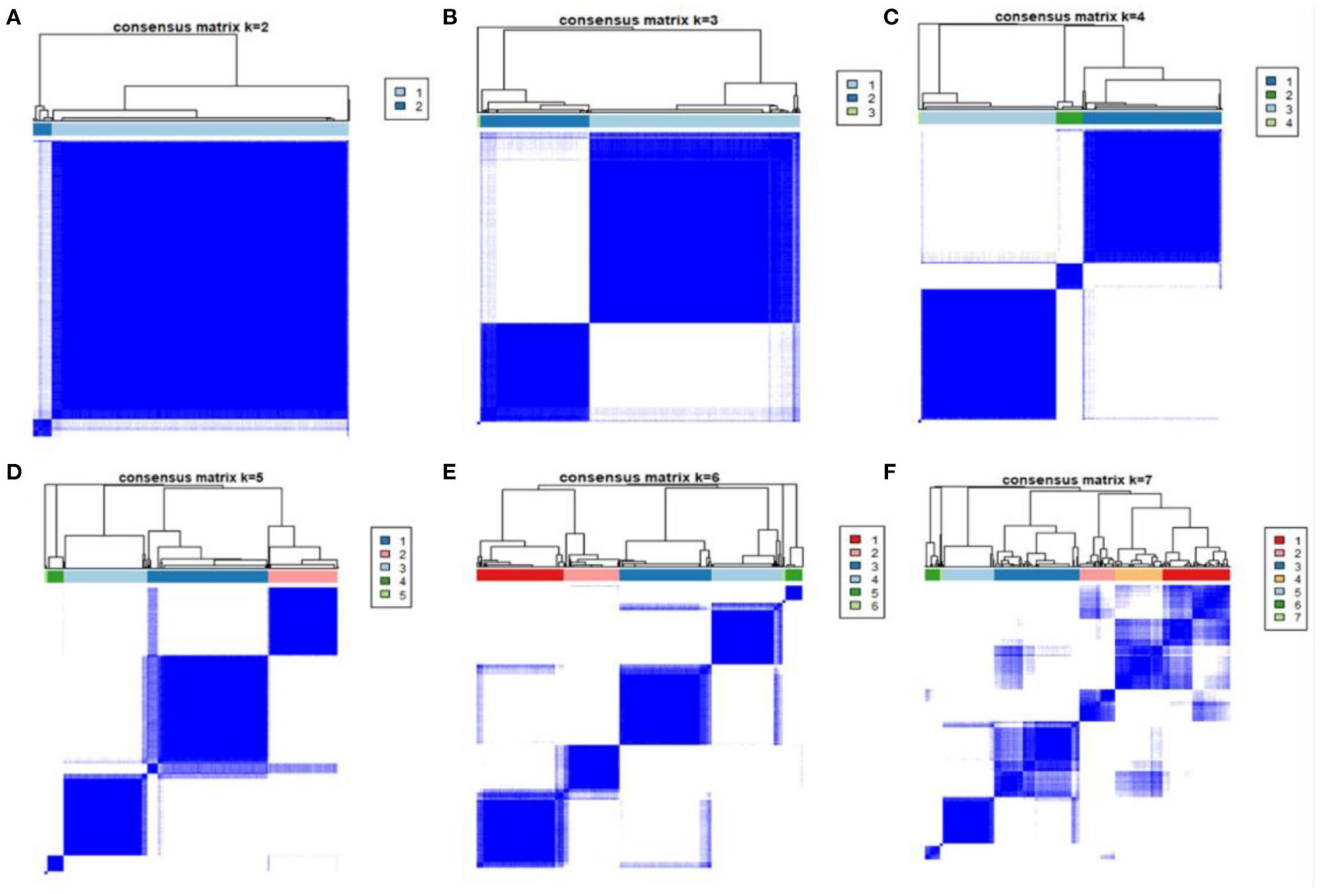
Consensus matrix heatmaps of consensus *k*-means clustering. Consensus matrix heatmaps of different subgroup numbers (*k* = 2, 3, 4, 5, 6, 7). When *k* = 3, the model exhibited the clearest separation of the consensus matrix heatmap.

The patients were classified as 2 phenotypes to 7 phenotypes through unsupervised clustering analysis, shown in (Figures 1A–F). As the 4-class model showed the clearest separation of the matrix heatmap (Figure 1), 4 phenotypes were utilized in the current study. The numbers of patients in Phenotypes I, II, III and IV were 319, 169, 492 and 11, respectively.

**Figure 2:**
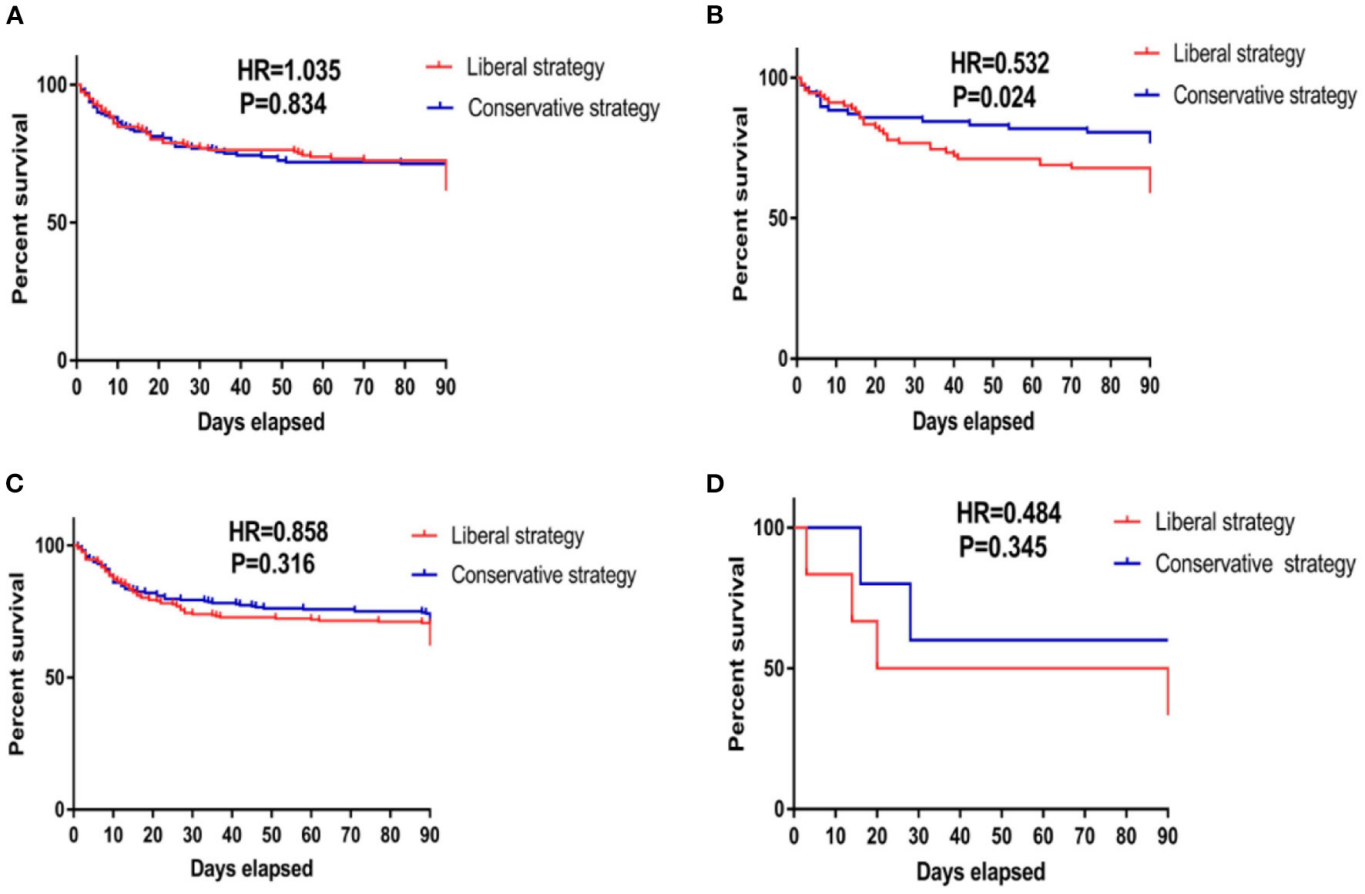
Kaplan–Meier survival curves of 90-day cumulative mortality for patients receiving conservative strategy and liberal strategy among those with the four phenotypes. **(A)** The survival curves of 90-day cumulative mortality of patients classified as phenotype I. The Kaplan–Meier survival analysis indicated that compared with the liberal strategy, conservative fluid management could not improve the 90-day mortality in patients classified as phenotype I (HR = 1.035, *P* = 0.843). **(B)** The survival curves of 90-day cumulative mortality of patients classified as phenotype II. The Kaplan–Meier survival analysis indicated that compared with the liberal strategy, conservative fluid management significantly improved 90-day mortality in patients classified as phenotype II (HR = 0.532, *P* = 0.024). **(C)** The survival curves of 90-day cumulative mortality of patients classified as phenotype III. The Kaplan–Meier survival analysis indicated that compared with the liberal strategy, conservative fluid management could not improve the 90-day mortality in patients classified as phenotype III (HR = 0.858, *P* = 0.316). **(D)** The survival curves of 90-day cumulative mortality of patients classified as phenotype IV. The Kaplan–Meier survival analysis indicated that compared with the liberal strategy, conservative fluid management could not improve the 90-day mortality in patients classified as phenotype IV (HR = 0.484, *P* = 0.345). HR, hazard ratio.

Phenotype II was identified as the specific population that benefited from the conservative fluid-management strategy because the conservative fluid-management strategy significantly improved the 90-day cumulative mortality compared with the liberal fluid-management strategy (HR = 0.532, *P* = 0.024), as shown in Figure 2. Regarding secondary outcomes, the conservative fluid management strategy markedly decreased the 90-day mortality compared with the liberal fluid management strategy (25.3 vs. 41.1%, *P* = 0.030). In addition, the conservative fluid management strategy was superior to the liberal fluid management strategy in avoiding superinfection (10.10 vs. 14.40%, *P* = 0.037) and returned to assisted breathing (4.60 vs. 16.20%, *P* = 0.030), as shown in Table 1.

**Table 1:** Secondary outcomes in phenotype 2.

**Outcomes**	**Conservative strategy (*n* = 90)**	**Liberal strategy (*n* = 79)**	***P***
28 day mortality (%)	16.50%	23.30%	0.268
60 day mortality (%)	20.30%	32.50%	0.197
90 day mortality (%)	25.30%	41.10%	**0.030**
Unassisted breathing (%)	17.70%	24.40%	0.290
Super infection (%)	10.10%	14.40%	**0.037**
Returned to assisted breathing (%)	4.60%	16.20%	**0.030**
Hospital free days to 90 (day)	51.75 ± 43.50	48.12 ± 34.97	0.503
ICU free days to day 90 (day)	62.69 ± 31.91	57.73 ± 34.22	0.097
Ventilator free days to day 90	61.40 ± 35.24	63.27 ± 32.75	0.310

Patients with other phenotypes given the different fluid management strategies did not show a significant difference in clinical outcomes, as shown in Figure 2 and Table 1.

### Features of the Phenotypes

For better insight into the characteristics of the phenotypes, features among different phenotypes were compared and evaluated. Phenotype IV was not selected as the main observational cohort due to the small sample size.

Patients classified as phenotype II exhibited a higher WBC (20487.51 ± 7223.86/mm^3^) and had a higher incidence of anasarca (8.3%) and incidence of shock (26.6%) at baseline, as shown in Tables 2, 3. Therefore, phenotype II was defined as the “hyperinflammatory anasarca” phenotype. Other characteristics of phenotypes are illuminated in Tables 2, 3.

**Table 2:** Dichotomous characteristics in different phenotypes.

**Id**	**Phenotype 1 (*n* = 319)**	**Phenotype 2 (*n* = 169)**	**Phenotype 3 (*n* = 492)**	**Phenotype 4 (*n* = 11)**	***P***
Male sex	52.3%	56.8%	53.9%	63.6%	0.674
Shock	6.6%	8.3%	2.8%	9.1%	**0.011**
Surgery	4.1%	3.0%	6.1%	0.0%	0.294
Ethanol	9.1%	11.2%	13.4%	18.2%	0.302
**ARDS risk factor**					
Pneumonia	45.5%	48.5%	48.2%	63.4%	0.187
Sepsis	22.3%	18.9%	25.8%	9.1%	0.385
Trauma	8.5%	7.7%	6.7%	9.1%	0.874
Multiple transfusion	0.9%	0	1.0%	9.1%	0.223
Aspiration	15.7%	15.4%	14.6%	9.1%	0.483
Others	6.6%	9.5%	5.5%	0	0.304
**Anamnesis**					
AIDS	6.0%	4.7%	9.1%	9.1%	0.200
Leukemia	1.9%	0.6%	3.5%	9.1%	0.085
Lymphoma	0.6%	0.6%	2.0%	0.0%	0.282
Solid tumor	2.2%	0.0%	1.8%	0.0%	0.289
Immune suppression	4.7%	9.5%	10.6%	9.1%	**0.037**
Anasarca	16.0%	26.6%	17.7%	18.2%	**0.027**
Heart failure	4.7%	4.7%	2.8%	9.1%	0.343
Hypertension	32.9%	34.3%	28.5%	18.2%	0.224
Myocardial infarction	6.9%	5.9%	4.9%	0.0%	0.510
Dementia	2.2%	3.6%	2.2%	0.0%	0.713
Stroke	4.1%	3.6%	5.1%	0%	0.732
Hepatic failure	0.9%	0.5%	1.0%	0.0%	0.953
Cirrhosis	2.2%	2.4%	4.5%	0.0%	0.270
Peptic ulcer	5.6%	4.7%	3.0%	9.1%	0.238
Diabetes	18.8%	18.9%	17.5%	0.0%	0.409

**Table 3:** Continuous variables in different phenotypes.

**Id**	**Phenotype 1**	**Phenotype 2**	**Phenotype 3**	**Phenotype 4**	***P***
Age (year)	48.57 ± 16.15	53.86 ± 15.96	50.50 ± 15.88	47.82 ± 16.88	0.391
Height (cm)	169.14 ± 11.53	169.46 ± 10.57	170.19 ± 10.23	175.56 ± 5.54	0.059
Weight	83.32 ± 24.57	81.96 ± 23.07	81.91 ± 22.53	89.92 ± 18.89	0.588
Temperature (°C)	37.4 ± 0.9	37.4 ± 0.9	37.6 ± 1.1	37.6 ± 1.4	0.136
Systolic BP (mmHg)	113.19 ± 20.88	113.47 ± 19.86	114.09 ± 23.10	119.82 ± 19.21	0.751
Diastolic BP (mmHg)	59.58 ± 12.13	59.31 ± 12.78	59.30 ± 13.15	62.64 ± 12.43	0.847
Mean arterial pressure (mmHg)	77.17 ± 14.04	77.22 ± 13.79	77.04 ± 14.49	81.92 ± 13.50	0.737
Heart rate (bpm)	102.05 ± 20.42	100.02 ± 20.10	103.17 ± 21.83	98.09 ± 19.71	0.349
Respiratory rate (breaths/min)	34.31 ± 9.43	34.66 ± 9.03	35.14 ± 10.67	35.55 ± 17.15	0.712
CVP (mm H_2_O)	11.79 ± 4.61	11.89 ± 4.56	12.19 ± 4.90	11.55 ± 4.78	0.661
Urine output/24 h (ml)	1978.81 ± 1348.99	2150.12 ± 2063.27	2155.51 ± 1633.35	1908.27 ± 1212.69	0.448
Glasgow coma	10.59 ± 4.58	10.71 ± 4.50	10.91 ± 4.40	9.82 ± 4.24	0.689
PaO2 (mmHg)	92.40 ± 44.30	94.54 ± 43.55	96.04 ± 44.76	119.91 ± 74.04	0.191
PaO2/FiO2	125.93 ± 61.51	133.80 ± 66.92	133.84 ± 61.26	164.30 ± 77.28	0.091
Bicarbonate (mEq/L)	21.68 ± 5.43	21.67 ± 5.88	20.89 ± 5.61	21.00 ± 5.27	0.178
HCT (%)	32.74 ± 7.27	32.6 ± 6.69	32.29 ± 6.76	30.82 ± 4.51	0.677
Glucose (mg/dL)	142.92 ± 88.07	149.83 ± 79.36	135.78 ± 58.75	113.36 ± 20.87	0.080
Potassium (mEq/L)	3.98 ± 0.65	4.06 ± 0.66	3.98 ± 0.64	4.46 ± 0.60	**0.045**
Sodium (mEq/L)	138.74 ± 5.17	139.31 ± 6.86	138.88 ± 5.16	138.27 ± 3.74	0.717
WBC (/mm^3^)	17313.76 ± 10409.86	20487.51 ± 7223.86	8402.11 ± 9878.86	66836.36 ± 31126.13	**<0.001**
HGB (g/dL)	10.66 ± 2.04	10.63 ± 1.93	10.18 ± 1.81	10.05 ± 0.90	**0.001**
Platelets (1,000/mm^3^)	226.44 ± 123.08	244.66 ± 147.44	158.12 ± 106.95	135.36 ± 54.93	**<0.001**
Albumin (g/dL)	2.24 ± 0.66	2.16 ± 0.63	2.19 ± 0.62	2.50 ± 0.73	0.250
Bilirubin (mg/dL)	1.56 ± 2.86	1.48 ± 1.78	1.78 ± 4.21	3.76 ± 7.39	0.162
BUN (mg/dL)	22.74 ± 16.81	20.09 ± 23.37	25.25 ± 19.08	21.18 ± 10.85	0.025
Chloride (mEq/L)	107.43 ± 6.81	106.70 ± 8.32	108.15 ± 6.37	108.18 ± 5.74	0.100
Creatinine (mg/dL)	1.16 ± 0.77	1.28 ± 0.81	1.33 ± 0.92	1.14 ± 0.40	**0.039**
Total protein	5.11 ± 1.04	5.05 ± 0.99	4.95 ± 1.04	5.28 ± 0.94	0.136
APACHE III	95.72 ± 32.75	92.07 ± 27.18	93.99 ± 30.98	87.27 ± 25.56	0.551

*WBC, white blood cell count; HCT, red blood cell specific volume; HGB, hemoglobin concentration; BUN, blood urea nitrogen. Bold values mean P value less than 0.05*.

## Discussion

The fluid management strategy for ARDS is not very clear. The current secondary analysis of RCTs identified 4 ARDS phenotypes, and a conservative fluid management strategy significantly improved the 90-day mortality of patients classified as phenotype II compared with a liberal fluid management strategy. In addition, a conservative fluid-management strategy was superior to a liberal fluid-management strategy in avoiding superinfection and returned to assisted breathing. Phenotype II was defined as a “hyperinflammatory anasarca” phenotype due to the higher WBC count with the higher incidence of anasarca and incidence of shock at baseline.

The current study first found that patients exhibiting a “hyperinflammatory anasarca” phenotype could benefit from a conservative fluid management strategy. This specific population showed a higher WBC (20487.51 ± 7223.86/mm^3^) with a higher incidence of anasarca (8.3%) and incidence of shock (26.6%) at baseline. Distributive shock and oedema due to ARDS-induced systemic inflammatory host responses on cardiovascular systems were marked signs in these patients (16–19). Previous studies uncovered that oedema was an independent risk factor for superinfection. and the anasarca could increase the number of days of mechanical ventilation (2, 20, 21). Our analysis further demonstrated that relieving oedema through a conservative fluid management strategy could effectively avoid superinfection and return to assisted breathing in patients with phenotype II, which could be the main reason to explain why the conservative fluid management strategy improved the mortality of these ARDS populations. Meanwhile, in order to maintain mean arterial pressure ≥ 65 mmHg and sufficient cardiac output to achieve adequate tissue perfusion for important organs, vasopressors are critical and should be used early for patients classified as phenotype II. This strategy is also suggested by Surviving Sepsis Campaign guidelines for septic shock (22, 23).

Individual and detailed situations should be considered to select a suitable fluid management strategy in patients classified as having other phenotypes. As there were no significant differences in clinical outcomes between conservative and liberal fluid management strategies, the detailed therapies should depend on patients' individual morbid conditions. If shock-induced tissue hypoperfusion is a crucial clinical problem in certain patients, a conservative fluid management strategy should be cautiously used in these patients. However, if ARDS-induced shock is reversed, a conservative fluid-management strategy could be considered for the improvement of respiratory failure (24–26).

There are some limitations in this study: prospective validation is required before definitive conclusions regarding therapy can be drawn. Meanwhile, the study is also limited by the fact that the beneficial effect was not externally validated. In addition, specific populations who could benefit from liberal fluid management strategies or other therapeutic methods could not be identified in the current study.

## Conclusions

Patients exhibiting a “hyperinflammatory anasarca” phenotype could benefit from conservative fluid management strategies.

## Data Availability Statement

The original contributions presented in the study are publicly available. This data can be found here: ARDS network public database (http://www.ardsnet.org/).

## Ethics Statement

Ethical review and approval was not required for the study on human participants in accordance with the local legislation and institutional requirements. Written informed consent for participation was not required for this study in accordance with the national legislation and the institutional requirements. Written informed consent was not obtained from the individual(s) for the publication of any potentially identifiable images or data included in this article.

## Author Contributions

SZ took responsibility for the integrity and the accuracy of the data analysis. All authors contributed to the article and approved the submitted version.

## Conflict of Interest

The authors declare that the research was conducted in the absence of any commercial or financial relationships that could be construed as a potential conflict of interest.

## Publisher's Note

All claims expressed in this article are solely those of the authors and do not necessarily represent those of their affiliated organizations, or those of the publisher, the editors and the reviewers. Any product that may be evaluated in this article, or claim that may be made by its manufacturer, is not guaranteed or endorsed by the publisher.
