# Supplementary material for: ARDS Patients Exhibiting a “Hyperinflammatory Anasarca” Phenotype Could Benefit From a Conservative Fluid Management Strategy
Source: Front Med (Lausanne). 2021 Aug 25;8:727910. doi: 10.3389/fmed.2021.727910 (PMC8423915; doi:10.3389/fmed.2021.727910)
Supplement: Supplementary file 1 [file Data_Sheet_1.pdf]

Catalogue of Supplemental Material

Title	Content	Page
e-Table 1	Secondary outcomes in phenotype	2
e-Table2	Secondary outcomes in phenotype 3	3
e-Table 3	Secondary outcomes in phenotype 4	4
e-Table 4	Baseline in phenotype 1	5
e-Table 5	Baseline in phenotype 2	6
e-Table 6	Baseline in phenotype 3	7
e-Table 7	Baseline in phenotype 4	8
e-Figure 1	The survival curves of 4 phenotypes in ARDS	9

e-Table 1. Secondary outcomes in phenotype 1

**Outcomes**	**Conservative Strategy (n=158)**	**Liberal Strategy (n=161)**	**P**
**28 day mortality (%)**	23.4%	23.6%	0.969
**60 day mortality (%)**	27.2%	28.6%	0.788
**90 day mortality (%)**	39.2%	37.3%	0.718
**Unassisted breathing (%)**	22.2%	21.7%	0.929
**Super infection (%)**	20.2%	17.4%	0.311
**Returned to assisted breathing (%)**	13.8%	13.6%	0.940
**Hospital free days to 90(day)**	47.76±33.44	50.01±34.53	0.554
**ICU free days to day 90(day)**	58.46±33.53	60.29±34.08	0.630
**Ventilator free days to day 90**	60.39±34.12	63.60±34.40	0.404

e-Table2. Secondary outcomes in phenotype 3

Outcomes	Conservative Strategy (n=243)	Liberal Strategy (n=258)	P
**28 day mortality (%)**	25.9%	21.3%	0.225
**60 day mortality (%)**	28.4%	24.8%	0.364
**90 day mortality (%)**	38.3%	34.1%	0.333
**Unassisted breathing (%)**	24.7%	20.5%	0.268
**Super infection (%)**	30.0%	25.6%	0.266
**Returned to assisted breathing (%)**	13.7%	14.1%	0.891
**Hospital free days to 90(day)**	49.61±33.33	48.40±34.51	0.141
**ICU free days to day 90(day)**	60.13±32.71	58.43±34.90	0.059
**Ventilator free days to day 90**	63.15±33.50	60.52±35.23	0.055

e-Table 3. Secondary outcomes in phenotype 4

Outcomes	Conservative Strategy (n=6)	Liberal Strategy (n=5)	P
**28 day mortality (%)**	50.0%	40.0%	0.769
**60 day mortality (%)**	50.0%	40.0%	0.769
**90 day mortality (%)**	66.7%	40.0%	0.427
**Unassisted breathing (%)**	33.3%	60.0%	0.428
**Super infection (%)**	16.7%	20.0%	0.900
**Returned to assisted breathing (%)**	50%	33.3%	0.789
**Hospital free days to 90(day)**	32.57±34.77	40.75±47.09	0.401
**ICU free days to day 90(day)**	24.57±33.26	41.75±48.22	0.942
**Ventilator free days to day 90**	32.57±41.04	42.25±48.79	0.362

e-Table 4. Baseline in phenotype 1

Characteristic	Conservative Strategy (n=158)	Liberal Strategy (n=161)	P
**Age (year)**	49.23±16.04	47.92±16.28	0.470
**Male sex (%)**	89	77	0.129
**Primary lung injury (%)**			
**Pneumonia**	79	66	0.461
**Sepsis**	35	38	0.298
**Aspiration**	23	27	0.933
**Trauma**	13	14	0.263
**Multiple transfusions**	1	2	0.545
**other**	7	14	0.893
**Coexisting conditions (%)**			
**Diabetes**	30	30	0.935
**HIV infection or AIDS**	12	7	0.222
**Cirrhosis**	6	1	0.053
**Solid tumors**	3	4	0.722
**Leukemia**	2	2	0.424
**Lymphoma**	1	1	0.989
**Immunosuppression**	6	9	0.451
**Shock (%)**	11	10	0.787
**APACHE III score**	94.88±33.79	96.55±31.78	0.654
**Hemodynamic variables**			
**Mean arterial pressure (mm Hg)**	78.455±14.47	75.90±13.51	0.104
**CVP (cm H2O)**	12.08±4.63	11.51±4.59	0.268
**PaO2 (mm Hg)**	91.92±42.33	92.87±46.28	0.848

e-Table 5. Baseline in phenotype 2

Characteristic	Conservative Strategy (n=90)	Liberal Strategy (n=79)	P
**Age (year)**	50.15±15.68	49.52±16.35	0.796
**Male sex (%)**	50	46	0.728
**Primary lung injury (%)**			
**Pneumonia**	43	39	0.496
**Sepsis**	20	12	0.237
**Aspiration**	12	14	0.698
**Trauma**	8	5	0.377
**Multiple transfusions**	0	0	0.926
**other**	7	9	0.158
**Coexisting conditions (%)**			
**Diabetes**	17	15	0.987
**HIV infection or AIDS**	5	3	0.594
**Cirrhosis**	1	3	0.254
**Solid tumors**	0	0	NA
**Leukemia**	1	0	0.350
**Lymphoma**	1	0	0.350
**Immunosuppression**	8	8	0.785
**Shock (%)**	5	9	0.172
**APACHE III score**	94.88±33.78	96.55±31.78	0.655
**Hemodynamic variables**			
**Mean arterial pressure (mm Hg)**	76.95±13.75	77.54±13.91	0.787
**CVP (cm H2O)**	11.51±5.03	12.34±3.94	0.238
**PaO2 (mm Hg)**	91.24±44.03	98.30±42.96	0.294

e-Table 6. Baseline in phenotype 3

Characteristic	Conservative Strategy (n=243)	Liberal Strategy (n=258)	P
**Age (year)**	49.39±15.99	51.55±15.74	0.127
**Male sex (%)**	128	137	0.924
**Primary lung injury (%)**			
**Pneumonia**	112	125	0.261
**Sepsis**	68	59	0.291
**Aspiration**	32	40	0.865
**Trauma**	15	18	0.438
**Multiple transfusions**	1	4	0.453
**other**	15	12	0.217
**Coexisting conditions (%)**			
**Diabetes**	40	46	0.685
**HIV infection or AIDS**	18	27	0.232
**Cirrhosis**	10	12	0.770
**Solid tumors**	2	7	0.111
**Leukemia**	7	10	0.539
**Lymphoma**	6	4	0.463
**Immunosuppression**	25	27	0.948
**Shock (%)**	7	7	0.909
**APACHE III score**	92.27±31.46	95.59±30.50	0.240
**Hemodynamic variables**			
**Mean arterial pressure (mm Hg)**	77.55±15.28	76.55±13.72	0.436
**CVP (cm H2O)**	12.01±4.79	12.36±5.01	0.417
**PaO2 (mm Hg)**	93.23±40.43	98.67±48.42	0.174

e-Table 7. Baseline in phenotype 4

Characteristic	Conservative Strategy (n=6)	Liberal Strategy (n=5)	P
**Age (year)**	47.75±26.47	47.85±11.15	0.871
**Male sex (%)**	4	3	0.840
**Primary lung injury (%)**			
**Pneumonia**	6	1	0.297
**Sepsis**	0	1	0.375
**Aspiration**	6	4	0.297
**Trauma**	6	4	0.297
**Multiple transfusions**	6	4	0.297
**other**	0	0	NA
**Coexisting conditions (%)**			
**Diabetes**	0	0	NA
**HIV infection or AIDS**	1	0	0.389
**Cirrhosis**	0	0	NA
**Solid tumors**	0	0	NA
**Leukemia**	0	1	0.296
**Lymphoma**	0	0	NA
**Immunosuppression**	0	1	0.296
**Shock (%)**	1	0	0.389
**APACHE III score**	91.25±23.35	85.00±28.28	0.118
**Hemodynamic variables**			
**Mean arterial pressure (mm Hg)**	80.50±13.96	82.71±14.29	0.037
**CVP (cm H2O)**	10.00±2.16	12.42±5.76	0.661
**PaO2 (mm Hg)**	151.75±99.66	101.71±55.74	0.806

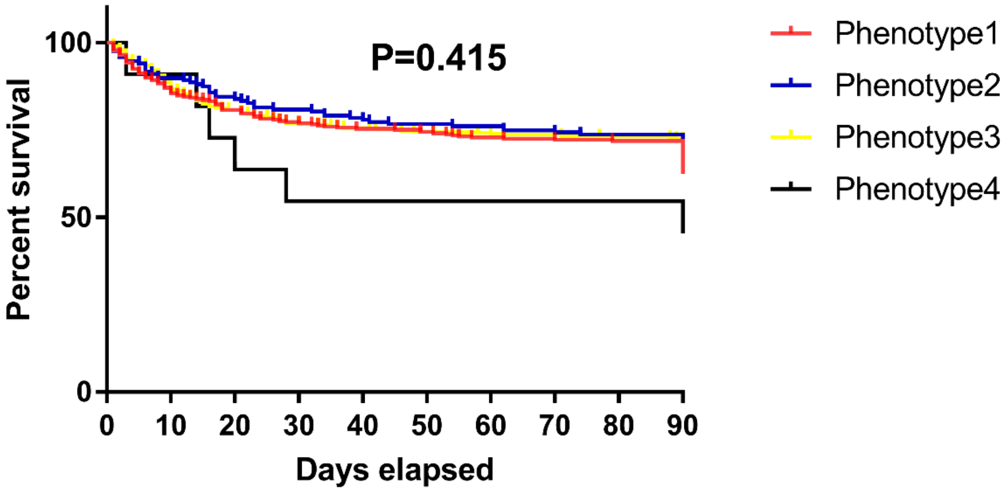

e-Figure 1. The survival curves of 4 phenotypes in ARDS
